# Association between body mass index and endometriosis risk: a meta-analysis

**DOI:** 10.18632/oncotarget.14916

**Published:** 2017-01-31

**Authors:** Liu Yong, Zhang Weiyuan

**Affiliations:** ^1^ Department of Gynecology, Beijing Obstetrics and Gynecology Hospital, Capital Medical University, Beijing, China; ^2^ Department of Obstetrics and Gynecology, Beijing Obstetrics and Gynecology Hospital, Capital Medical University, Beijing, China

**Keywords:** body mass index, obesity, overweight, endometriosis, meta-analysis

## Abstract

**Background:**

Epidemiological studies have sought to establish a relationship between a woman's current body mass index and endometriosis, but with varying results. This meta-analysis was to summarize the current epidemiological evidence.

**Methods:**

Pertinent studies were identified by searching PubMed and Web of Science through November 2016. Study-specific risk estimates were combined using fixed or random effects models depending on whether significant heterogeneity was detected.

**Results:**

A total of 11 studies (two cohort studies and nine case-control studies) was included in the meta-analysis. The pooled relative risk of endometriosis was 0.67 (95% CI: 0.53, 0.84) for each 5 kg/m^2^ increase in current body mass index, with statistical significant heterogeneity across the studies (*P* <0.001, I^2^ =86.9%). Compared with normal weight women, the pooled relative risk for obese women was 0.89 (95% CI: 0.83, 0.96), which was lower than that for overweight women (relative risk =0.97; 95% CI: 0.91, 1.05). The combined estimate was robust across subgroup and sensitivity analyses and no observed publication bias was detected.

**Conclusion:**

This study suggested that higher body mass index may be associated with lower risk of endometriosis. Further work will need to focus on elucidating underlying biologic mechanism that contribute to the initiation of endometriosis.

## INTRODUCTION

Endometriosis is a common gynecological inflam-matory disease with a prevalence of 6~10% in the general female population [1]. It can cause pelvic inflammation, adhesions, infertility and chronic pain [1]. It is estimated that annual costs of endometriosis have exceed $49 billion in the United States [2]. Despite its significant impact on the health-care system, the risk factors of endometriosis remain poorly elucidated.

A number of biologic risk factors, such as a taller height or lesser weight, have been reported to be associated with risk of endometriosis [1, 3–8]. However, most remain inconclusive as risk factors of endometriosis. During the past decades, epidemiological studies [3–7, 9–13] have sought to establish a relationship between a woman's current body mass index (BMI) and endometriosis, but with varying results. Some studies [5, 9–13] indicated an inverse association between endometriosis and BMI, but others [3, 4, 6] did not observe such relation. Herein, we conducted a meta-analysis to quantitatively assess this association.

## RESULTS

### Literature search

**Figure 1:**
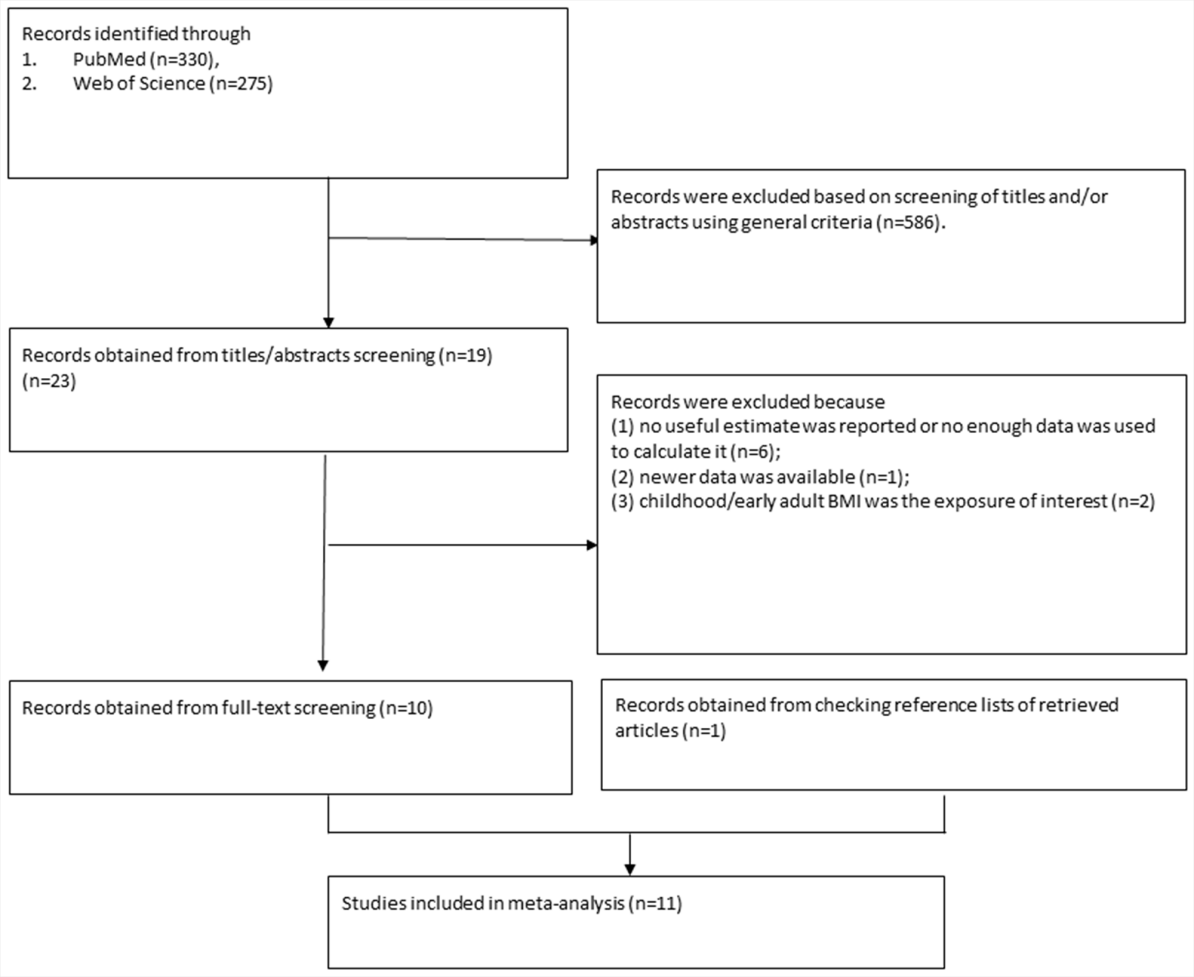
References searched and selection of studies in the meta-analysis

A total of 3403 records were identified after the search of the databases. After the title and abstract screening, 3380 records which did not meet the pre-specified inclusion criteria were excluded. After the full-text screening, 12 articles [14–25] did not meet the inclusion criteria, of which nine [14–22] did not have useful estimate, two [23, 24] used childhood/early adult BMI as the exposure of interest, and one [25] was an updated study. Moreover, we identified one additional publication were after manually searching the reference lists of selected studies [8]. Finally, a total of 11 publications [3–13] were included in present meta-analysis(Figure 1).

### Description of the studies

There were nine case-control studies [3, 5–9, 11–13] and two cohort studies [4, 10] included in the meta-analysis. Nine studies [3–5, 7, 9–13] reported, or provided necessary data to calculate the study-specific RRs and their corresponding 95% CIs for each 5 kg/m^2^ increase in BMI associated with endometriosis; five studies [6, 8–10, 12] reported the study-specific RRs and 95% CIs of endometriosis for overweight women compared with normal weight women; and three studies [8–10] reported the study-specific RRs and 95% CIs for obese women compared with normal weight women(Table 1).

**Table 1:** Characteristics of studies included in the meta-analysis, 2003 to 2016

Study	Year	Location	Design	Study population	Age	Exposure measurement	Outcome evaluation	No. of Cases	No. of controls/cohort size	Matching/confounding factors
Shahbazi	2016	Iran	Case-control study	Fertile women	Mean age: case=36.20; controls=39.96	Measured byinterviewer	Medical examination	46	53	Ethnicity, area of residence, age, smoking, length of menstrual cycle, menstrual blood flow, gravidity and parity
Ashrafi	2016	Iran	Case-control study	Infertile women	16-46 years	Measured byinterviewer	Examined by laparoscopy	341	332	NA
Upson	2016	USA	Case-control study	Cases and controls were from healthcare system	18–49 years	Interviewed in person	Histologic confirmation	310	727	Age
Shah	2013	USA	Cohort study	Female nurses	25–42 years	Measured byinterviewer	Examined by laparoscopy	5504	116,430	Parity, race, ethnicity, birth weight, age at menarche, length of menstrual cycle, pattern of menstrual cycle, age at first birth, time since last birth, current alcohol use, current smoking status, infertility, use of oral contraceptives, and perceived body size at ages 5 and 10 years
Peterson	2013	USA	Cohort study	Population cohort	18-44 years	Anthropometric assessment	MRI-visualized & Histologically confirmed	14	127	Age and site
Moini	2013	Iran	Case-control study	Infertile women	Mean age: case=30; controls=31	Questionnaire	Histological confirmation	250	153	NA
Hediger	2005	USA	Case-control study	Women scheduled for laparoscopy	18–45 years	Self-reported	Examined by laparoscopy	32	52	Age, early menarche, age at first sexual activity, height, parous
Ferrero	2005	Italy	Case-control study	Women with benign gynaecological conditions	Mean age: case=33.3; controls=33.7	Record linkage	Examined by laparoscopy	366	248	Age, presence of severe pelvic pain/dysmenorrhea, oral contraceptive use, infertility, and previous live births
Parazzini	2004	Italy	Case-control study	Controls were women with acute non-gynaecological, non-hormonal, non-neoplastic conditions	aged <65 years	Structured questionnaire	Examined by laparoscopy	504	498	Age, study, calendar year at interview
Hemmings	2004	Canada	Case-control study	Women scheduled for laparoscopy or laparotomy	Mean age=37.3	Structured questionnaire	laparoscopy or laparotomy	1881	890	Gravidity, education, time lag between first pregnancy and menarche, length of menses, and presence of leiomyoma
Signorello	2003	USA	Case-control study	Cases were infertility-associated endometriosis; Control group A included fertile women without endometriosis; Control group B included infertile women without endometriosis.	23 to 44 years	self-administered	Examined by laparoscopy	50	89 (Control group A); 47(Control group B)	Age, education level, menstrual cycle, and exercise

The included studies were conducted in the North America (n=6) [4, 6–8, 10, 11], Europe (n=2) [12, 13], and Asia (n=3) [3, 5, 9]. There was a total of 9,298 women diagnosed with endometriosis. All the endometriosis cases were diagnosed and confirmed by medical examination, such as examined by laparoscopy. The quality scores ranged from 5 to 8 with a median value of 7. There were six studies [7–12] that had ≥7 awarded points, which were defined as high-quality studies (Supplementary Tables 1 and 2).

### Overall and subgroup results

**Figure 2:**
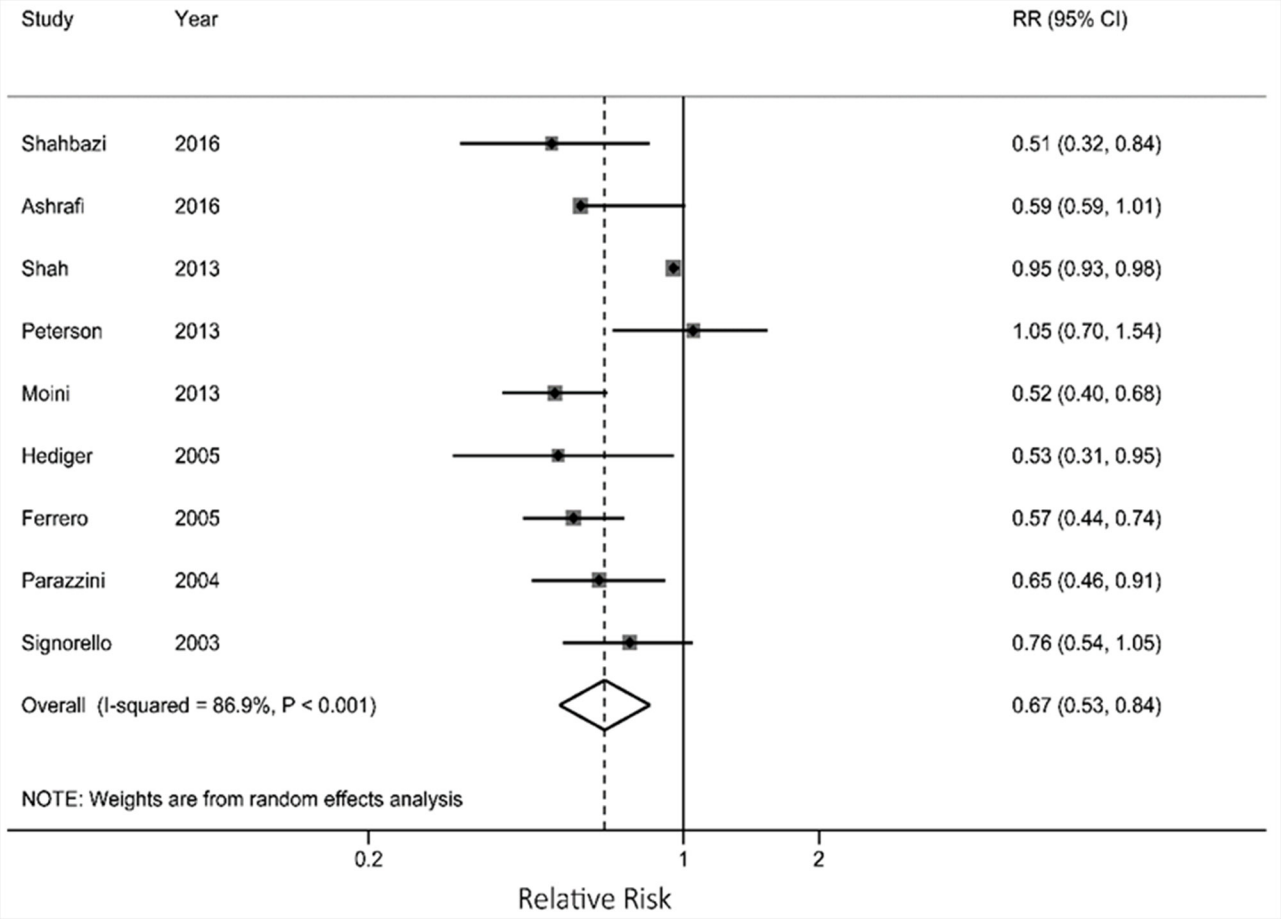
Results from meta-analysis for each 5 kg/m^2^ increase in current body mass index associated with endometriosis risk

As shown in Figure 2, the overall analysis showed a 33% reduction in the risk of endometriosis for each 5 kg/m^2^ increase in BMI (RR=0.67; 95% CI: 0.53, 0.84), with statistical significant heterogeneity across the studies (*P* <0.001, I^2^ =86.9%). No indication of publication bias was detected by Begg's test (P = 0.602).

**Figure 3:**
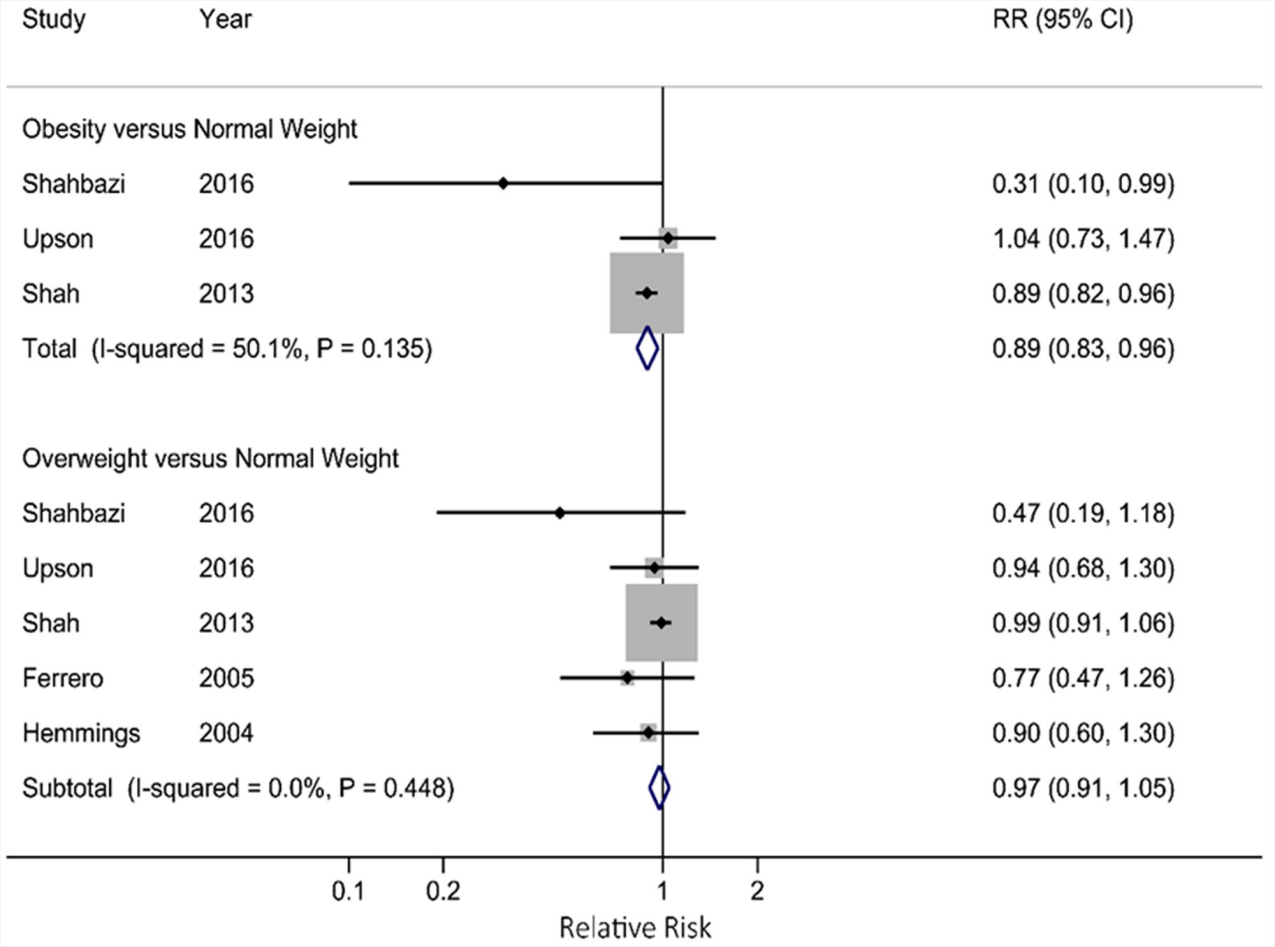
Forest plot for associations of obesity and overweight with endometriosis risk

Compared with normal weight women, the pooled RR for obese women was 0.89 (95% CI: 0.83, 0.96), which was lower than that for overweight women (RR=0.97; 95% CI: 0.91, 1.05) (Figure 3). When we stratified by study design, the pooled RR was 0.95 (95% CI: 0.93, 0.98) for cohort studies, which was higher than that for case-control studies (RR=0.59; 95% CI: 0.52, 0.66). When we restricted the analysis in infertile women, the pooled RR was 0.68 (95% CI: 0.51, 0.89), which was lower than that for fertile women (RR=0.75; 95% CI: 0.39, 1.44). When stratified by study location, studies conducted in North America (RR=0.95; 95% CI: 0.92, 0.97), Europe (RR=0.60; 95% CI: 0.49, 0.74) and Asia (RR=0.55; 95% CI: 0.46, 0.65) all showed significantly inverse association between BMI and endometriosis risk. The strength of the associations attenuated when we pooled the studies adjusted for smoking status, length of menstrual cycle, and age at menarche (Table 2). In sensitivity analyses, we recalculated the pooled RRs by sequentially excluding one study. The eight study-specific RRs ranged from a low of 0.63 (95% CI: 0.54, 0.73) to a high of 0.69 (95% CI: 0.55, 0.87) after omission of Shah et al. and Moini et al., respectively. The results were similar when the analysis was restricted to the high-quality studies (RR=0.67; 95% CI: 0.49, 0.92).

**Table 2:** Summary estimates and corresponding 95% confidence intervals for association of endometriosis risk with each 5 kg/m^2^ increase in body mass index

Subgroup by study characteristics	No. of studies	PooledRR	95% CI	I^2^ (%)	*P_H_*^1^	*P_H_*^2^
Overall analysis	9	0.67	0.53, 0.84	86.9	<0.001	
Study design						<0.001
Case-control study	7	0.59	0.52, 0.66	0	0.678	
Cohort study	2	0.95	0.93, 0.98	0	0.619	
Study location						0.004
North America	4	0.95	0.92, 0.97	51.0	0.106	
Europe	2	0.60	0.49, 0.74	0	0.548	
Asia	3	0.55	0.46, 0.65	0	0.761	
Study population						0.595
Infertile women	4	0.68	0.51, 0.89	83.3	<0.0001	
Fertile women	2	0.75	0.39, 1.44	86.6	0.006	
Number of cases						0.725
<250	4	0.71	0.51, 0.98	55.1	0.083	
≥250	5	0.65	0.47, 0.89	91.9	<0.001	
Exposure measurement						0.175
Measured by interviewers	4	0.76	0.55, 1.05	83.8	<0.001	
Self-reported or others	5	0.60	0.52, 0.69	0	0.459	
Study quality						0.857
High	5	0.67	0.49, 0.92	85.0	<0.001	
Low	4	0.66	0.50, 0.86	65.4	0.034	
**Adjustment for potential confounding factors**						
Smoking status						0.349
Yes	2	0.73	0.40, 1.33	84.3	0.012	
No	7	0.64	0.54, 0.75	44.5	0.094	
Length of menstrual cycle						0.234
Yes	3	0.77	0.56, 1.06	75.2	0.018	
No	6	0.62	0.52, 0.74	46.4	0.097	
Parity						0.923
Yes	4	0.64	0.43, 0.96	88.0	<0.001	
No	5	0.67	0.54, 0.84	58.7	0.046	
Oral contraceptive use						0.698
Yes	3	0.69	0.44, 1.06	89.3	<0.001	
No	6	0.65	0.53, 0.80	52.8	0.006	
Age at menarche						0.224
Yes	2	0.76	0.43, 1.33	76.0	0.041	
No	7	0.63	0.54, 0.75	46.2	0.084	

^1^P value for heterogeneity within each subgroup.

^2^P value for heterogeneity between subgroups in meta-regression analysis.

## DISCUSSION

To our knowledge, this is the first meta-analysis to assess the relationship between a woman's current BMI and endometriosis risk. Here, we observed a significant inverse association, which suggested a possible reduced risk of endometriosis for women with higher BMI. The combined estimate was robust across sensitivity analyses.

The natural history of endometriosis remains unknown. Previous study reported that women with more advanced stage of endometriosis trend to have an even lower BMI compared with women with milder disease [11]. Of note, two studies reported an inverse association between childhood body size and risk of endometriosis [23, 24], which was in agreement with the observed association of adult BMI and endometriosis risk. Further elucidation of the role of body size over critical windows may be helpful to better understand the disease.

Several important issues should be taken into further considerations in this study. First, regarding the utilization of BMI, most genetic and molecular effects upon body weight are likely to become obscured. It is argued that clinical categories of the BMI may not provide enough etiological information to reflect the nature of obesity. Other new concept such as “adiposopathy” which was defined as adipose tissue dysfunction will have to be included in any future studies using BMI to associate to endometriosis. Second, it has been suggested that the inverse relationship between obesity and endometriosis is due to diagnostic bias. Obese women with pelvic pain may be less likely to be suggested with an operative intervention, which may subsequently lower the possibility of a laparoscopic diagnosis of endometriosis.

Some limitations should also be taken into considerations. First, residual confounding inherent in the original studies may distort the association between BMI and endometriosis. However, major potential confounders, such as smoking, parity, age at menarche and length of menstrual cycle, were adjusted in most of the original studies. Second, misclassification of BMI may occur, especially for studies with a self-reported height and weight. However, subgroup analysis stratified by exposure measurement method showed similar results between studies with height and weight measured by interviewers and by self-reported. Third, there is a significant heterogeneity across the studies. The observed heterogeneity may originate from various sources, such as different study designs and different study populations. Fourth, although publication bias was not observed in present meta-analysis, the statistical power for publication bias test was lower when the number of studies was limited.

In conclusion, this study suggested that higher BMI may be associated with lower risk of endometriosis. Large cohort studies are needed to confirm this inverse association. Further studies are also need to unveil the underlying biologic mechanism concerning the development of endometriosis.

## MATERIALS AND METHODS

### Study selection

We followed standard criteria for conducting and reporting of meta-analyses of observational studies [26]. We performed a computerized literature search through November 2016 using the following key words in PubMed and Web of Knowledge: (“body mass index” OR “obesity” OR “overweight” OR “underweight” OR “weight” OR “anthropometric” OR “body size” OR “body figure”) AND (“endometriosis”). The identified publications were reviewed independently for their relevance to the research topic by two authors. We also manually searched the reference lists of relevant publications to identify additional studies. A set of pre-specified inclusion criteria was applied during the review, and discrepancies were resolved by consensus. To be included in the meta-analysis, studies had to: 1) report current BMI as the exposure of interest, 2) report endometriosis as the outcome of interest, 3) use a cohort, case-cohort or case-control design, 4) provide estimates of relative risk (RR), hazard ratio, or odds ratio with confidence intervals (CIs) or standard errors or the data necessary to calculate these.

Because endometriosis is a rare outcome in general female population, the odds ratio in a case-control study is approximate equal to a rate ratio, hazard ratio or relative risk in a cohort study. Thus, we used the relative risk to measure the association between BMI and endometriosis risk. If multiple estimates were provided, priority was given to the multivariable-adjusted risk estimates that were adjusted for the most potential confounding factors in original studies. If more than one study was conducted in the same population, the most recent report was selected for our analysis.

### Data extraction

We used a standardized reporting form to abstract the following data from each publication: the first author's name, the year of publication, the country in which the study was conducted, the age of the study population, the size of the cohort or number of controls, the number of cases, the assessment method of current BMI, the categories of BMI, the ascertainment of the endometriosis and the RRs and 95% CIs for endometriosis risk associated with those categories, and the potential confounders which were adjusted in multivariable models.

### Quality assessment

To assess study quality, a 9-point system on the basis of the Newcastle-Ottawa Scale [27] was used in which a study was judged on 3 broad categories for case-control studies and cohort studies as follows: the selection of study groups, comparability of groups, and ascertainment of either the exposure or outcome of interest. The high-quality study was defined as one with ≥7 points which is a median value of all the included studies.

### Statistical analysis

Based on the World Health Organization classification, BMI was categorized into underweight (<18.5 kg/m^2^), normal weight (18.5–24.9 kg/m^2^), overweight (25–29.9 kg/m^2^) and obese (≥30 kg/m^2^). To examine the association between BMI and endometriosis risk, we pooled the study-specific RR for each 5 kg/m^2^ increase in current BMI. For the studies in which only categorical results were reported, we used the method proposed by Greenland and Longnecker [28] and Orsini et al. [29] to calculate the trend in RR per 5 kg/m^2^ increase in current BMI. We also calculated the pooled RRs of endometriosis for obese or overweight women compared with normal weight women. We used a fixed effect model to pool the study specific estimates unless significant heterogeneity was observed, then the random effect model proposed by DerSimonian and Laird was used [30].

For further assessing the association of BMI with endometriosis risk, we conducted analyses stratified by study design, study location, exposure assessment method, number of cases, and study quality. We also conducted subgroup analyses stratified by whether the studies adjusted for potentially important confounders or important risk factors, including smoking status, length of menstrual cycle, parity, oral contraceptive use, and age at menarche. In addition, we performed a sensitivity analysis of the influence of individual studies on the summary estimate by repeating the meta-analysis excluding one study at a time.

Heterogeneity among studies was assessed with the Q and I2 statistics, and results were defined as heterogeneous for a P value < 0.10 or an I2 > 50% [31]. Publication bias were evaluated by visual inspection of funnel plot and formal testing by using Begg's tests [32].

Statistical analyses were conducted using Stata, version 14.0 (StataCorp LP, College Station, Texas). Two-sided P values <0.05 were considered statistically significant.

## SUPPLEMENTARY MATERIALS TABLES


