# Supplementary material for: Association between body mass index and endometriosis risk: a meta-analysis
Source: Oncotarget. 2017 Jan 31;8(29):46928–36. doi: 10.18632/oncotarget.14916 (PMC5564533; doi:10.18632/oncotarget.14916)
Supplement: Supplementary file 1 [file oncotarget-08-46928-s001.pdf]

Association between body mass index and endometriosis risk: a meta-analysis

SUPPLEMENTARY MATERIALS

Supplementary Table 1: Quality of case-control studies included in the meta-analysis[1]

First author	Adequate definition of cases	Representativeness of cases	Selection of control subjects	Definition of control subjects	Controls for important factor or additional factors[2]	Exposure assessment	Same method of as certainment for all subjects	Non responserate[3]	Total quality score
Shahbazi	1	1	0	1	2	1	1	1	8
Upson	1	1	1	1	1	0	1	1	7
Ashrafi	1	1	0	1	0	1	1	1	6
Moini	1	1	0	1	0	0	1	1	5
Hediger	1	1	0	1	2	0	1	1	7
Ferrero	1	1	0	1	2	0	1	1	7
Parazzini	1	1	0	1	1	0	1	1	6
Hemmings	1	1	0	1	1	0	1	1	6
Signorello	1	1	0	1	2	0	1	1	7

[1]A study could be awarded a maximum of one star for each item except for the item "Control for important factor or additional factor".
[2]A maximum of 2 stars could be awarded for this item. Studies that controlled for age received one star, whereas studies that controlled for most of the other important confounders such as length of menstrual cycle, oral contraceptive use, parity and age at menarche received an additional star.
[3]One star was assigned if there was no significant difference in the response rate between control subjects and cases by using the chi-square test ($P < 0.05$).

Supplementary Table 2: Quality of cohort studies included in the meta-analysis[1]

First author	Representativeness of the exposed cohort	Selection of the non-exposed cohort	Ascertainment of exposure	Outcome of interest was not present at start of study	Controls for important factor or additional factors[2]	Assessment of outcome	Follow-up long enough for outcomes to occur[3]	Adequacy of follow up of cohorts[4]	Total quality score
Shah	0	1	1	1	2	1	1	1	8
Peterson	0	1	1	1	1	1	0	1	6

[1]A study could be awarded a maximum of one star for each item except for the item "Controls for important factor or additional factors."
[2]A maximum of 2 stars could be awarded for this item. Studies that included adjustment for age received one star, and studies that included most of the other important confounders such as length of menstrual cycle, oral contraceptive use, parity and age at menarche received an additional star.
[3]A cohort study with a follow-up time >10 years was assigned one star.
[4]A cohort study with a follow-up participation rate >80% was assigned one star.
